# Comparative *de novo* transcriptome profiles in *Asparagus officinali*s and *A*. *kiusianus* during the early stage of *Phomopsis asparagi* infection

**DOI:** 10.1038/s41598-017-02566-7

**Published:** 2017-06-01

**Authors:** Mostafa Abdelrahman, Naoyuki Suzumura, Mai Mitoma, Satoshi Matsuo, Takao Ikeuchi, Mitsutaka Mori, Kyoko Murakami, Yukio Ozaki, Masaru Matsumoto, Atsuko Uragami, Akira Kanno

**Affiliations:** 1 Graduate School of Life Sciences, Tohoku University, Katahira 2-1-1, Aoba-ku, Sendai 980-8577 Japan; 2 Botany Department, Faculty of Science, Aswan University, Aswan, 81528 Egypt; 3 Institute of Vegetable and Floriculture Science, National Agriculture and Food Research Organization (NARO), 360 Kusawa, Ano, Tsu, Mie, 514-2392 Japan; 4 Kagawa Prefectural Agricultural Experiment Station, 1534-1 Ayagawa, Ayauta, Kagawa 761-2306 Japan; 5 Faculty of Agriculture, Kyushu University, Fukuoka, 811-2307 Japan; 6 Institute of Tropical Agriculture, Kyushu University, Fukuoka, 812-8581 Japan; 7 Institute of Vegetable and Floriculture Science, NARO, Tsukuba, Ibaraki, 305-8519 Japan

## Abstract

*Asparagus kiusianus*, an important wild relative of cultivated asparagus (*A*. *officinalis*), exhibits resistance to stem blight disease caused by *Phomopsis asparagi*. However, the mechanisms underlying this resistance are not understood and no transcriptomic or genetic resources are available for this species. *De novo* transcriptome sequencing of *A*. *officinalis* and *A*. *kiusianus* stems was performed 24 h after inoculation with *P*. *asparagi*. In total, 35,259 and 36,321 transcripts were annotated in *A*. *officinalis* and *A*. *kiusianus*, respectively. 1,027 up-regulated and 752 down-regulated transcripts were differentially expressed in the two *Asparagus* species. RNA sequencing data were validated using quantitative real-time reverse transcription PCR. Several defense-related genes including *peroxidase 4*, *cationic peroxidase SPC4*-*like*, *pathogenesis*-*related protein*-*1*-*like*, and jasmonic acid biosynthesis and signaling-related genes including *phospholipase D alpha 1*, *12*-*oxophytodienoate reductase* and *jasmonate*-*induced protein 23 KD* were up-regulated in *A*. *kiusianus* relative to *A*. *officinalis*. In addition, infected *A*. *kiusianuns* exhibited a substantial increase in jasmonic acid and methyl jasmonate relative to *A*. *officinalis*. Peroxidase activity was significantly elevated in infected *A*. *kiusianus* compared with infected *A*. *officinalis*. Our transcriptomic database provides a resource for identifying novel genes and molecular markers-associated with *Phomopsis* disease resistance and will facilitate breeding and improvement of cultivated asparagus varieties.

## Introduction

*Asparagus officinalis* L., a dioecious species of the family Asparagaceae, is an economically important horticultural crop worldwide because of its culinary and medicinal properties. The total worldwide asparagus production in 2013 was approximately 7.95 million tonnes, of which 89.4% was produced in Asia, 7.2% in the Americas, 3.2% in Europe, and 0.2% in Oceania^1^. Stem blight disease caused by *P*. *asparagi* is the most serious disease for asparagus production in many parts of the world, including Japan, China, Australia, New Zealand, Italy, Greece, and the United States^2–4^. Disease symptoms are first seen as small light brown lesions on the lower part of the stem. The primary lesions subsequently become extended, forming larger dark brown oval-shaped lesions that eventually lead to complete stem desiccation and stem death^4, 5^. Stem blight disease is mainly controlled using expensive chemical fungicides. However, concerns have been raised regarding the human and environmental impacts of fungicides, as well as their impacts on the capacity of *P*. *asparagi* to survive in various environments. Therefore, production of new asparagus cultivars with strong resistance to stem blight disease has become an urgent need in the context of sustainable crop production.

*Asparagus* is a large genus comprising 200–300 species distributed across the Old World Continents^6^. Diverse ecological niches led to the development of an extensive variety of different *Asparagus* species with different morphological and physiological traits. Wild *Asparagus* species represent a potential genetic resource for the development of disease-resistant *Asparagus* germplasm with desirable physiological attributes. Several wild *Asparagus* species exhibited a strong disease resistance phenotype in previous studies, but production of interspecific hybrids by crossing with cultivated *A*. *officinalis* was hampered by the genetic distance between species^7^. *A*. *kiusianus* is a wild diploid (2n = 2x = 20) species endemic to the coastal region of the Sea of Japan in the Kyushu area of Japan^8^. Analysis of the non-coding region of chloroplast DNA indicated that *A*. *kiusianus* was genetically closer to *A*. *officinalis* than many other wild *Asparagus* species^9^, and interspecific hybrids and backcross progenies were successfully obtained between *A*. *kiusianus* and *A*. *officinalis* ^10^. Evaluation of stem blight disease severity in *A*. *kiusianus*, *A*. *officinalis*, and their interspecific F_1_ hybrids revealed strong disease resistance characteristics in *A*. *kiusianus* and the F_1_ hybrids compared with *A*. *officinalis* ^4^. Although the molecular and physiological mechanisms underlying stem blight disease resistance remain unknown, these findings suggest that wild *A*. *kiusianus* could play a significant role in improving stem blight disease resistance in *A*. *officinalis*. Further development of *Asparagus* genetic resources and research to discover novel disease resistance alleles will help to improve germplasm utilization and facilitate breeding of new asparagus varieties.

Next-generation sequencing (NGS) for large-scale transcriptome analysis has become the technique of choice for generating large amounts of expression data in a relatively short time. Gene expression data have provided insights into the processes underlying gene expression and have facilitated gene discovery^11–13^. To identify the broad transcriptional network-associated with *Phomopsis* disease resistance in *Asparagus*, we conducted transcriptome analysis of susceptible *A*. *officinalis* and resistant wild *A*. *kiusianus* stems 24 h after inoculation with *P*. *asparagi* or mock inoculation with sterile distilled water (SDW). High-throughput Illumina HiSeq 2500 technology was used, and high-quality reads were *de novo* assembled into unique transcripts, which were then comprehensively evaluated and annotated. Several common and unique genes that were differentially expressed (DEG) between susceptible and resistant *Asparagus* species as a consequence of *P*. *asparagi* infection were detected. Selected candidate genes were validated using quantitative real-time reverse transcription PCR (qRT-PCR). qRT-PCR and Kyoto Encyclopedia of Genes and Genomes (KEGG) pathway analyses of the DEG revealed many defense and stress-related genes including *peroxidase 4*, *cationic peroxidase SPC4*-*like*, *chitinase*-*6*, *pathogenesis*-*related protein 1*-*like*, and jasmonic acid (JA) biosynthesis and signaling-related genes are the major components of the *A*. *kiusianus* response to *P*. *aspargi* infection relative to *A*. *officinalis*. To dissect the significant role of JA pathway and other stress-related genes in *A*. *kiusianus* resistance, phytohormone accumulation including JA, methyl jasmonate (MeJA), salicylic acid (SA) and abscisic acid (ABA) in *A*. *kiusianus* and *A*. *officinalis* 24 h post-inoculation in comparison with SDW-treated control plants was carried out using liquid chromatography-tandem mass spectrometry (LC-MS/MS). In addition, enzyme assays for stress-related enzymes such as catalase (CAT, EC 1.11.1.6) and peroxidase (POX, EC 1.11.1.7) were performed. To our knowledge, RNA sequencing has not been used to examine *Asparagus*-*P*. *asparagi* interactions previously. Our transcriptome dataset is therefore a valuable and unique resource that will facilitate future functional genetics studies and molecular marker development for asparagus breeding.

## Results

### Stem blight disease occurrence in wild and cultivated *Asparagus* species

**Figure 1:**
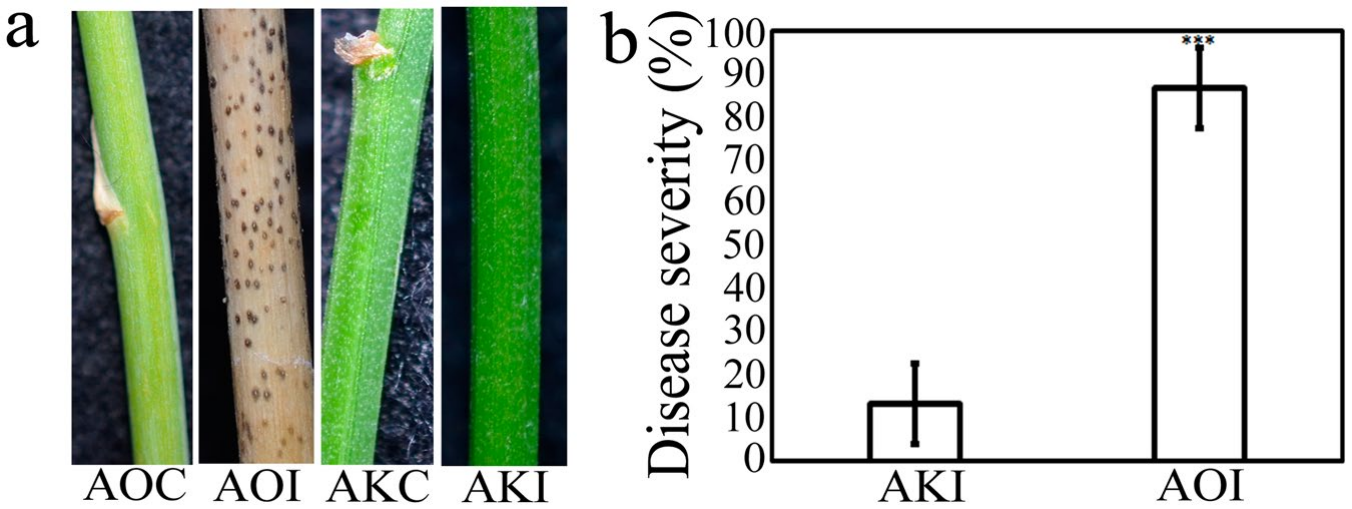
Stem blight disease symptoms in cultivated *Asparagus officinalis* and wild *A*. *kiusianus* 3 weeks after inoculation with *Phomopsis asparagi*. (**a**) Disease symptoms in *A*. *officinalis* treated with sterile distilled water (AOC), *A*. *officinalis* inoculated with *P*. *asparagi* (AOI), *A*. *kiusianus* treated with sterile distilled water (AKC), and *A*. *kiusianus* inoculated with *P*. *asparagi* (AKI). (**b**) Disease severity percentage in AKI and AOI plants. Values represent the mean ± standard error (SE) of three independent replicates (*n* = 3). Asterisks indicate significant difference between AOI and AKI plants as determined by Student’s *t*-test (***P < 0.001).

Differences in disease occurrence between susceptible cultivated *A*. *officinalis* ‘Mary Washington 500W’ and resistant wild *A*. *kiusianus* (AK0501 strain) were observed after inoculation with *P*. *asparagi* spores under greenhouse conditions. Primary signs of infection, seen as dark brown lesions were first visually detected on susceptible *A*. *officinalis* at 7 days post-inoculation. The fungus then spread through the stem, resulting in a fully diseased stem by 14 days post-inoculation (Fig. 1a). By contrast, typical disease symptoms were not seen on resistant *A*. *kiusianus* and the fungus was unable to spread (Fig. 1a), demonstrating that *A*. *kiusianus* exhibited resistance to *P*. *asparagi*. Disease severity was significantly (*P* < 0.001) different between the two *Asparagus* species, at 86.66% and 13.33% in *A*. *officinalis* and *A*. *kiusianus*, respectively (Fig. 1b).

### Transcriptome sequencing and *de novo* assembly

**Figure 2:**
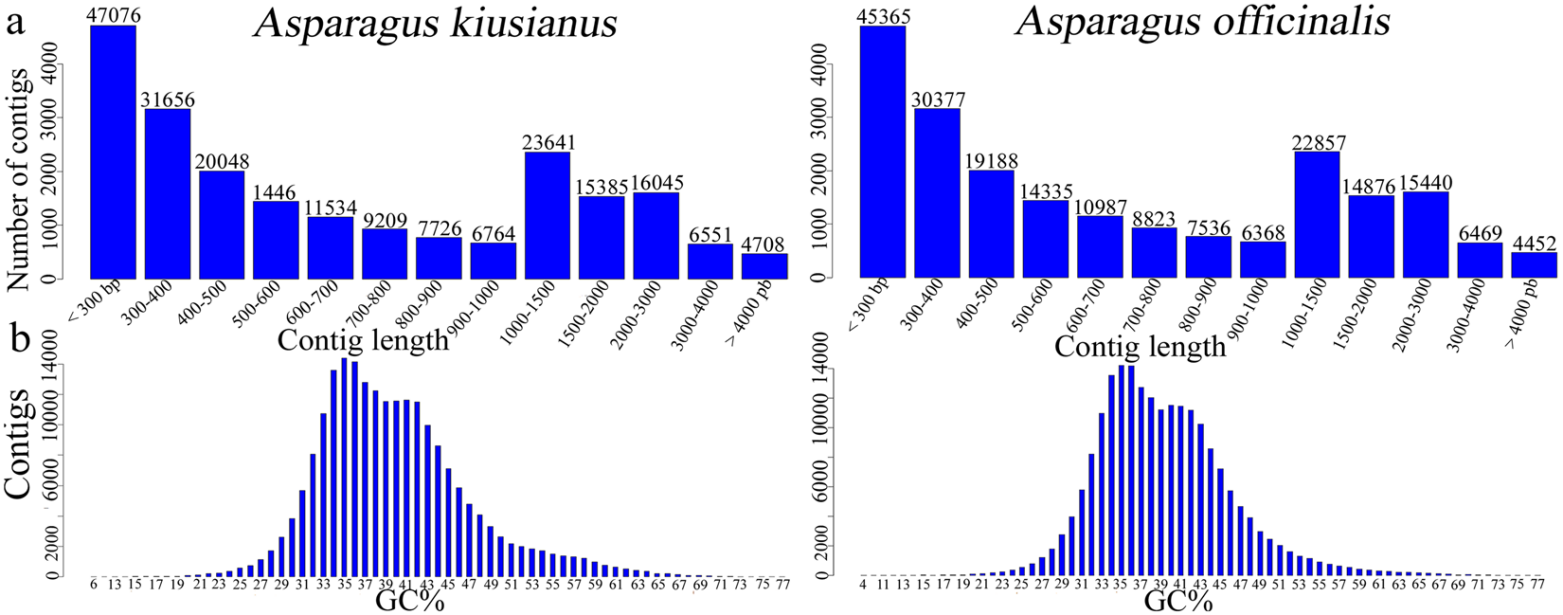
Transcript length and GC-content distribution graph in *Asparagus officinalis* and *A*. *kiusianus*. (**a**) Length distribution of assembled transcriptome fragments in *A*. *officinalis* and *A*. *kiusianus*. (**b**) GC-content percentage distribution in unique transcripts of *A*. *officinalis* and *A*. *kiusianus*.

To identify genes involved in the response to stem blight disease, stems of susceptible cultivated *A*. *officinalis* ‘Mary Washington 500W’ and resistant wild *A*. *kiusianus* (AK0501 strain) were inoculated with *P*. *asparagi*. Samples were collected from SDW-treated control stems and inoculated stems 24 h after infection. Four RNA-Seq libraries were generated: *A*. *officinalis* SDW-treated control (AOC), *A*. *officinalis*-inoculated (AOI), *A*. *kiusianus* SDW-treated control (AKC), and *A*. *kiusianus*-inoculated (AKI). Libraries were sequenced using an Illumina HiSeq 2500 platform. In total, 44,65 and 41,55 Gbp nucleotide sequence were generated from *A*. *officinalis* and *A*. *kiusianus*, respectively. After removing adapter sequences and low-quality sequences, 94,440,525; 101,235,105; 90,926,718; and 94,362,439 high-quality cleaned reads with 100 bp were obtained from AOC, AOI, AKC, and AKI, respectively. Details of the *Asparagus* transcriptome data are shown in Supplementary Table S1. The high-quality reads were *de novo* assembled into unique transcripts using Trinity software. In total, 206,164 and 213,950 assembled transcripts (average length 973 bp) were obtained from *A*. *officinalis* and *A*. *kiusianus*, respectively. Many transcripts were short; nevertheless, 64,094 and 66,330 transcripts were assembled from *A*. *officinalis* and *A*. *kiusianus*, respectively, that were >1,000 bp in length. The quality of transcriptome assemblies was assessed, and the length distribution of the assembled transcripts in both *Asparagus* species is shown in Fig. 2a. The GC-content of the assembled *Asparagus* transcripts was similar in both species, with a distribution peak at ~35% GC (Fig. 2b).

### Functional annotation of *Asparagus* transcriptomes

**Figure 3:**
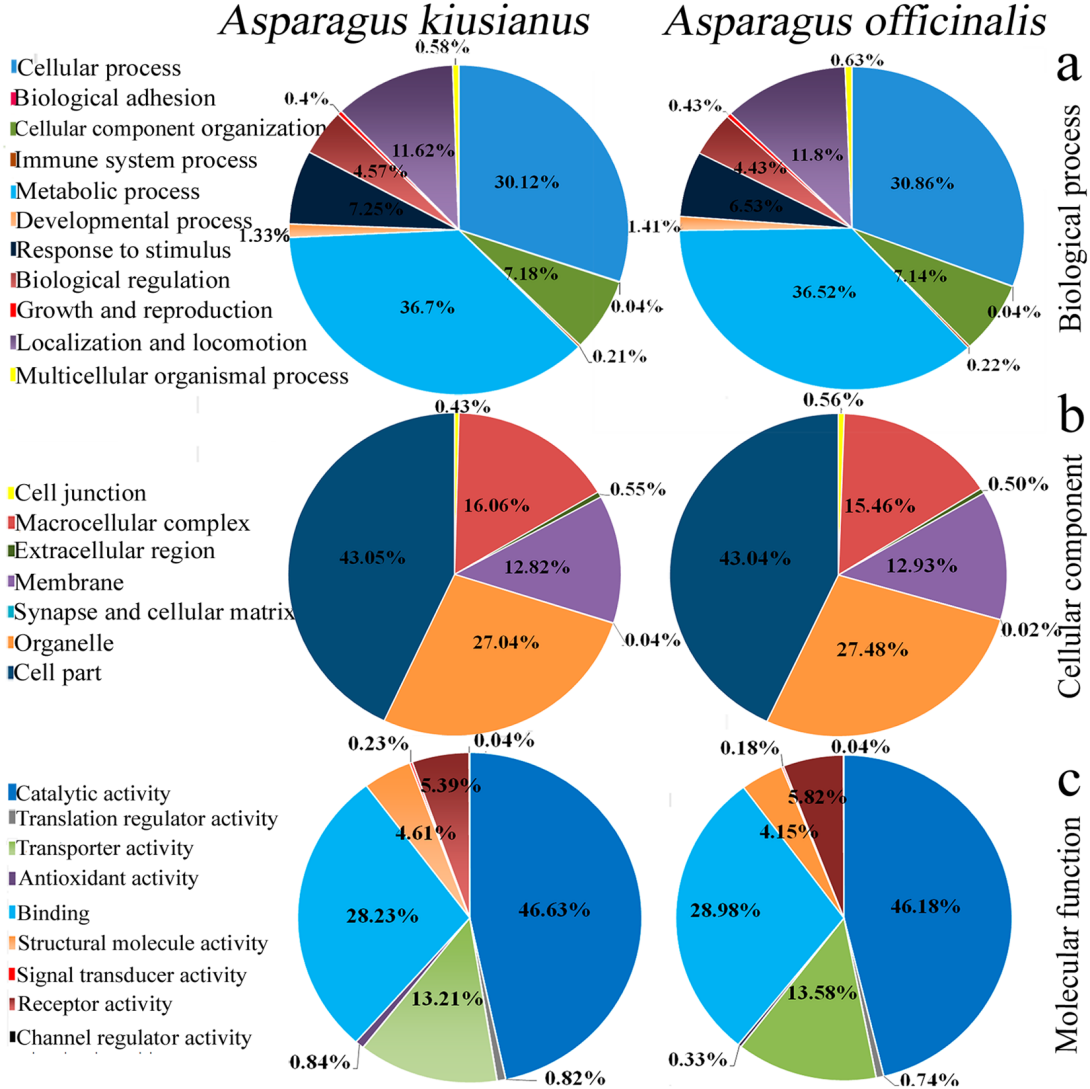
Pie chart of gene ontology (GO) analysis of *Asparagus officinalis* and *A*. *kiusianus*. Results are summarized for the three main GO categories: (**a**) biological process, (**b**) cellular component, and (**c**) molecular function.

To functionally annotate the assembled *Asparagus* transcripts, translated sequences were searched against the NCBI non-redundant proteins (nr) database (http://www.ncbi.nlm.nih.gov) and the UniProt (Swiss-Prot) protein database using Blastp. Of the assembled sequences, 35,259 translated transcripts in *A*. *officinalis* and 36,321 in *A*. *kiusianus* matched known proteins in Swiss-Prot. A relatively large proportion of the *Asparagus* translated transcripts had no hits to any known proteins. Gene Ontology (GO) terms were assigned to the *Asparagus* transcripts. In total, 22,148 and 24,411 transcripts from *A*. *officinalis* and *A*. *kiusianus*, respectively, were assigned at least one GO term. Of these, 10,006 and 11,027 were assigned for biological process, 5,026 and 5,591 were assigned for cellular component, and 7,116 and 7,793 were assigned for molecular function, for *A*. *officinalis* and *A*. *kiusianus*, respectively. Within the biological process category (Fig. 3a), the most abundant groups were metabolic process (GO: 0008152, 36%), cellular process (GO: 0009987, 30%), localization and locomotion (GO: 0051179, 11%), cellular component organization (GO: 0071840, 7%), and response to stimulus (GO: 0050896, 6%). Within the cellular component category, cell part (GO: 0044464, 43%), organelle (GO: 0043226, 27%), macrocellular complex (GO: 0032991, 16%), and membrane (GO: 0016020, 12%) were the most highly represented groups (Fig. 3b). Catalytic activity (GO: 0003824, 46%), binding (GO: 0005488, 28%), transporter activity (GO: 0005215, 13%), receptor activity (GO: 0004872, 5%), and structural molecule activity (GO: 0005198, 4%) were the most highly represented groups in the molecular function category (Fig. 3c).

### Differentially Expressed Genes (DEGs)

**Figure 4:**
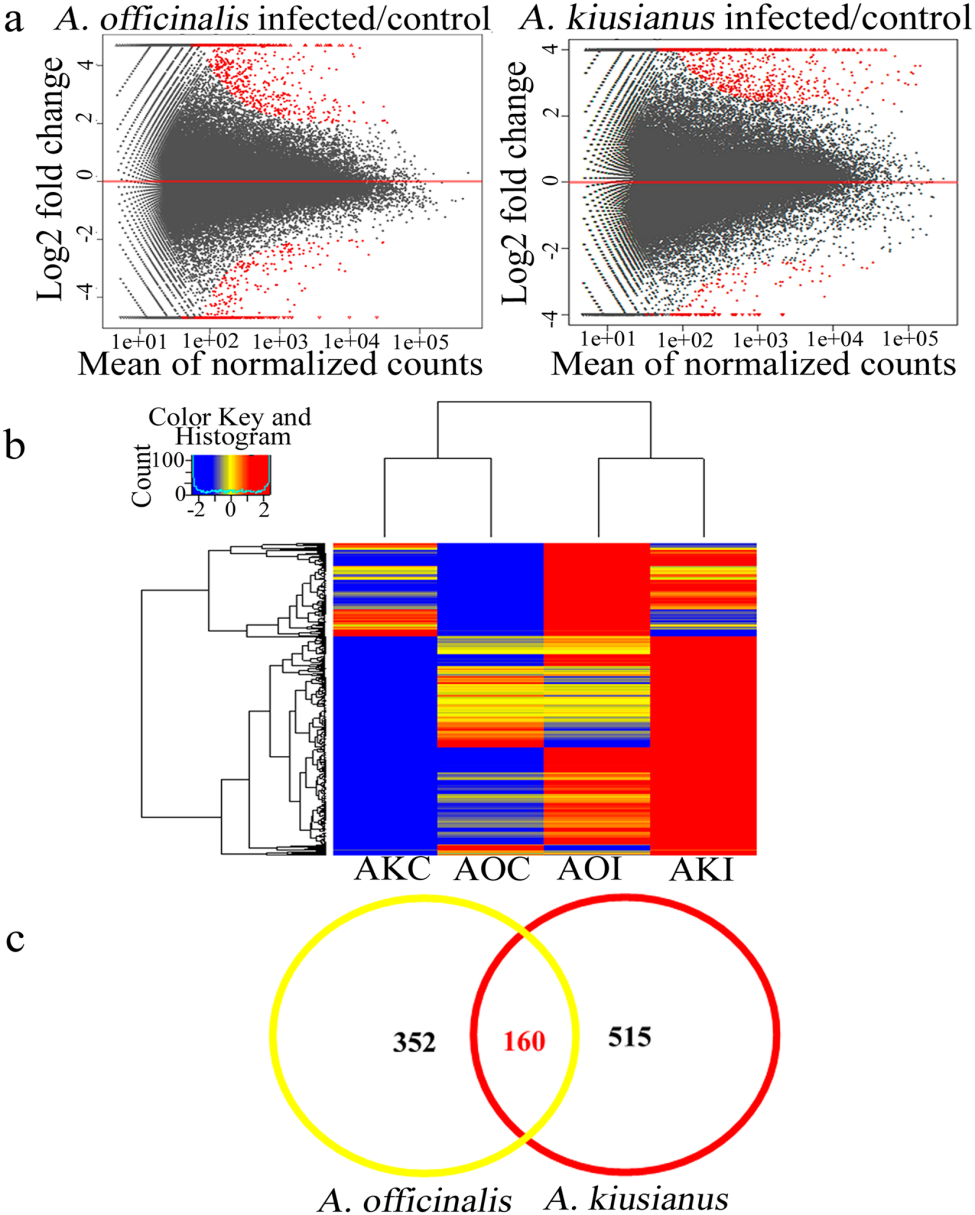
Differentially expressed genes (DEGs) in *Asparagus officinalis* and *A*. *kiusianus* stems 24 h after inoculation with *Phomopsis asparagi*. (**a**) MA scatter plot analysis showing log 2 fold change (y-axis) of pairwise comparisons between *A*. *officinalis* inoculated with *P*. *asparagi* (infected) versus *A*. *officinalis* treated with sterile distilled water (control), and *A*. *kiusianus* inoculated with *P*. *asparagi* (infected) versus *A*. *kiusianus* treated with sterile distilled water (control). DEGs are highlighted in red. (**b**) Heatmap clustering of up-regulated transcripts (fold change ≥ 2) in *A*. *officinalis* inoculated with *P*. *asparagi* (AOI) relative to *A*. *officinalis* treated with sterile distilled water (AOC), and *A*. *kiusianus* inoculated with *P*. *asparagi* (AKI) relative to *A*. *kiusianus* treated with sterile distilled water (AKC). (**c**) Venn diagram of transcripts up-regulated in *A*. *officinalis* and *A*. *kiusianus* 24 h after inoculation with *P*. *asparagi*.

**Figure 5:**
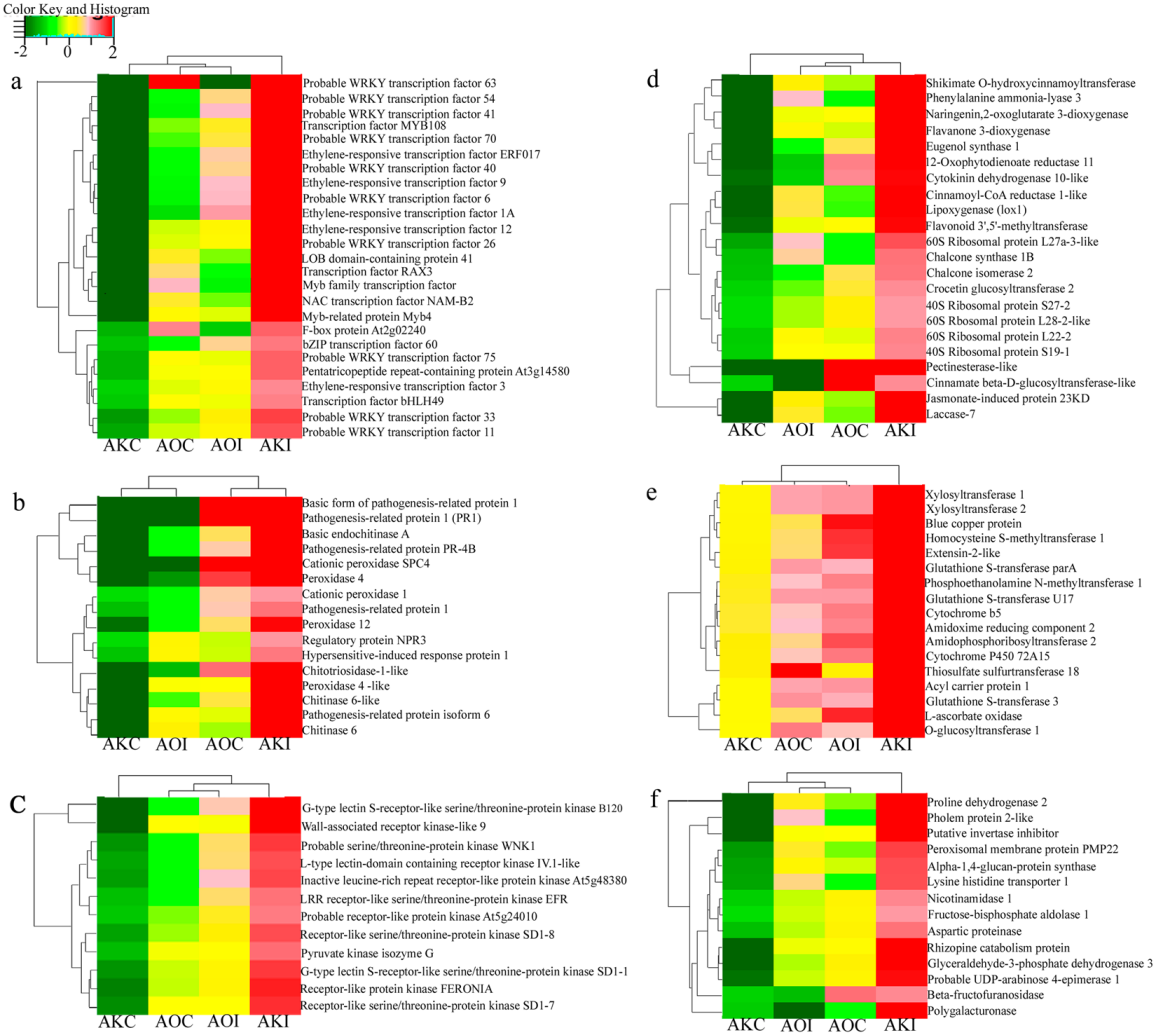
Heatmap clustering of most up-regulated (fold change ≥ 2) transcripts in *Asparagus kiusianus* inoculated with *Phomopsis asparagi* (AKI) and their relative expression levels in *A*. *officinalis* inoculated with *P*. *asparagi* (AOI). *A*. *kiusianus* and *A*. *officinalis* treated with sterile distilled water (AOC and AKC, respectively) served as controls. Genes are categorized according to those encoding (**a**) transcription factors, (**b**) Defence response factors, (**c**) kinase signaling proteins, (**d**) phenylpropanoid and ribosomal proteins, (**e**) transferase-related proteins, and (**f**) amino acid- and carbohydrate-related proteins.

Gene expression profiles were derived from the RNA-Seq data, and normalized DEGs were determined. To find genes that were induced by stem blight disease, pairwise comparisons were made between AOI and AOC and between AKI and AKC. A total of 1,779 differentially expressed transcripts were identified, of which 1,027 were up-regulated and 752 were down-regulated, in the two *Asparagus* species after inoculation with *P*. *asparagi* (Fig. 4a). Of the 1,027 up-regulated transcripts, 515 were up-regulated only in *A*. *kiusianus*, 352 were up-regulated only in *A*. *officinalis*, and 160 were up-regulated in both *Asparagus* species (Fig. 4b,c). These results demonstrated that the gene expression responses to *P*. *asparagi* infection differed between the two *Asparagus* species. GO term enrichment analysis indicated that genes involved in a range of biological processes such as transcriptional regulation, stress and defence response, protein kinase activity, phenylpropanoid and hormone biosynthesis and signaling, and cell wall assembly and organization were significantly enriched in the set of genes that was up-regulated in wild *A*. *kiusianus* but not in cultivated *A*. *officinalis* (Fig. 5).

In *A*. *kiusianus*, *WRKY6*, *WRKY33*, *WRKY40*, *WRKY41*, *WRKY*54, *WRKY63*, and *WRKY70* exhibited 5.45-, 2.94-, 6.32-, 9.46-, 11.28-, 11.20-, and 8.11-fold higher expression in inoculated (AKI) plants than in control (AKC) plants, respectively. By contrast, in *A*. *officinalis*, these *WRKY* TFs were expressed at low levels in inoculated (AOI) plants compared with control (AOC) plants (Fig. 5a). These findings suggest that WRKY TFs are likely to play an important role in regulating transcription in *A*. *kiusianus* in response to *P*. *asparagi* infection. In *A*. *kiusianus*, expression of stress and defence response-related genes, including *peroxidase 4*, *peroxidase 12*, *chitinase*-*6*, *chitotriosidase*-*1*-*like*, and *pathogenesis*-*related protein*-*1* (*PR*-*1*), was elevated in inoculated (AKI) plants, at levels 24.99-, 3.74-, 7.0-, 7.40-, and 114.2-fold higher, respectively, than seen in control (AKC) plants. By contrast, in *A*. *officinalis*, these genes were down-regulated in inoculated (AOI) plants relative to control (AOC) plants (Fig. 5b). In addition, the expression of JA biosynthesis-related genes including *phospholipase D alpha 1* (*PLDα1*), *lipoxygenase 1* (*LOX1*), *12*-*oxophytodienoate reductase 11* (*OPR*) and *Jasmonate*-*induced 23 KD protein* (*JIP*-*23 KD*) was evaluated in AKI plants at levels 2.22-, 5.25-, 6.02- and 36-fold higher expression than in AKC plants (Fig. 5d). However, these genes exhibited a significant reduction in AOI plants relative to AOC plants (Fig. 5d).

### Metabolic pathways by KEGG analysis of differentially up-regulated genes in *A*. *kiusianus*

To characterize the metabolic pathways of the identified DEG in *A*. *kiusianus*, gene classification was performed based on Kyoto Encyclopedia of Genes and Genomes (KEGG) database^14^. Pathway categories were listed in the Supplementary Table S3. A total of 32 pathways were significantly identified. Genes involved in metabolic pathways, biosynthesis of secondary metabolites, plant-pathogen interaction, kinase signaling pathway and plant hormone signal transduction were the most represented up-regulated DEG in *A*. *kiusianus* in response to *P*. *aspargi* (Supplementary Table S3).

### qRT-PCR

**Figure. 6:**
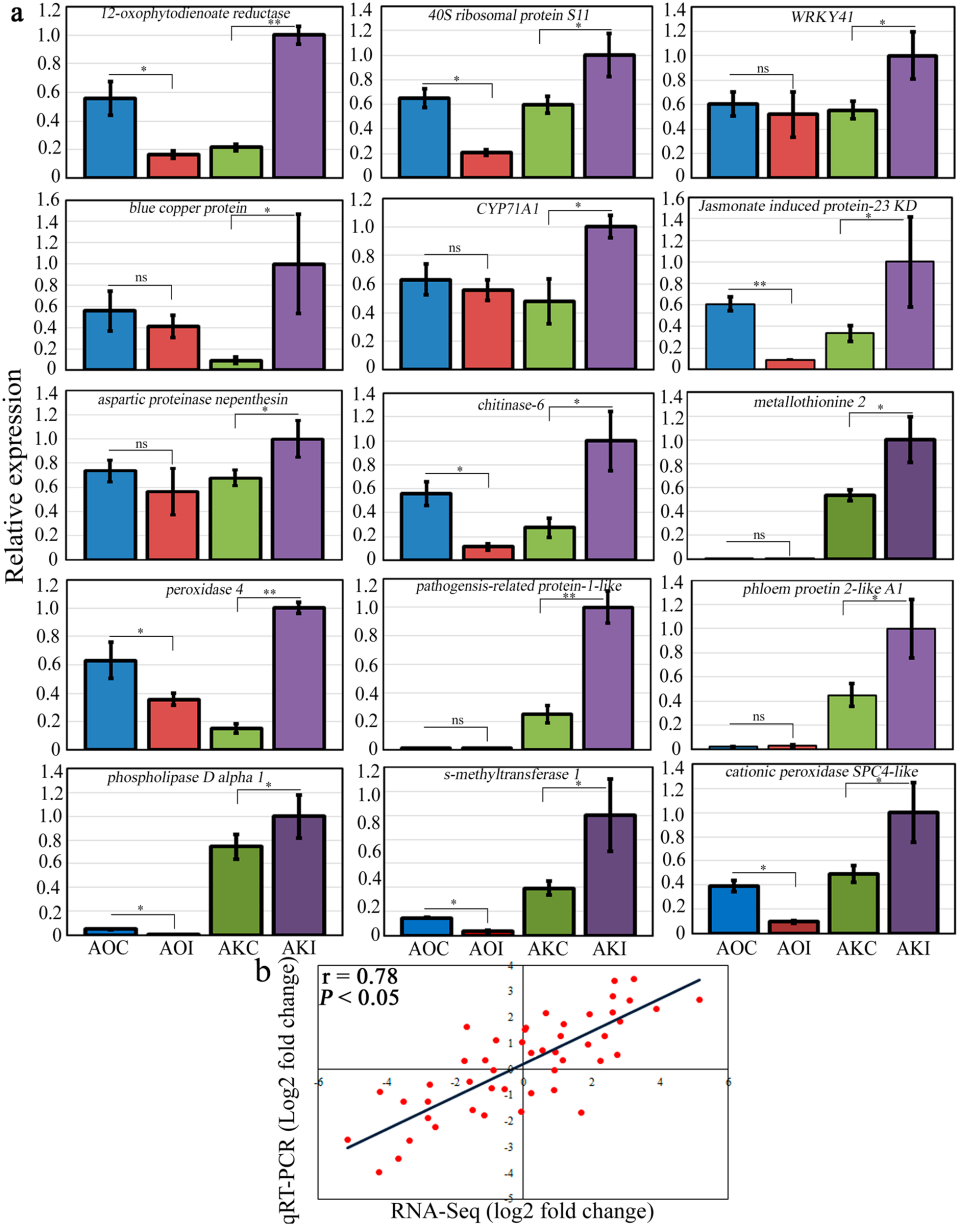
Validation of RNA-Seq data with quantitative real-time reverse transcription PCR (qRT-PCR). (**a**) Bar plot of qRT-PCR relative expression in *Asparagus kiusianus* and *A*. *officinalis* treated with distilled water (AKC and AOC, respectively) and inoculated with *Phomopsis asparagi* (AKI and AOI, respectively). (**b**) Scatter plot correlation between qRT-PCR and RNA-Seq log2 fold changes (Pearson’s correlation coefficient = 0.78). Values represent the mean on three independent biological replicates (n = 3) ± standard deviation (SD). Significance levels are given as: *(*P* < 0.05) and **(*P* < 0.01) and according to analysis of variance (ANOVA).

Fifteen up-regulated transcripts related to stress and defence, hormone biosynthesis and signaling, and ribosome were selected for validation of RNA-Seq data using qRT-PCR. Prior to qRT-PCR analysis, RT-PCR amplification was performed to confirm the suitability of the primer pairs. Single amplicons of the expected size were produced for each primer pair (Supplementary Fig. S1). The qRT-PCR results confirmed that the selected transcripts were all strongly up-regulated in AKI plants compared with AKC plants and down-regulated in AOI plants compared with AOC plants (Fig. 6a). In addition, the qRT-PCR results positively correlated (r = 0.78) with the RNA-Seq data (Fig. 6b), validating the sequencing results.

### Phytohormone accumulation in wild and cultivated *Asparagus* species 24 h post-inoculated with *P*. *asparagi*

**Figure 7:**
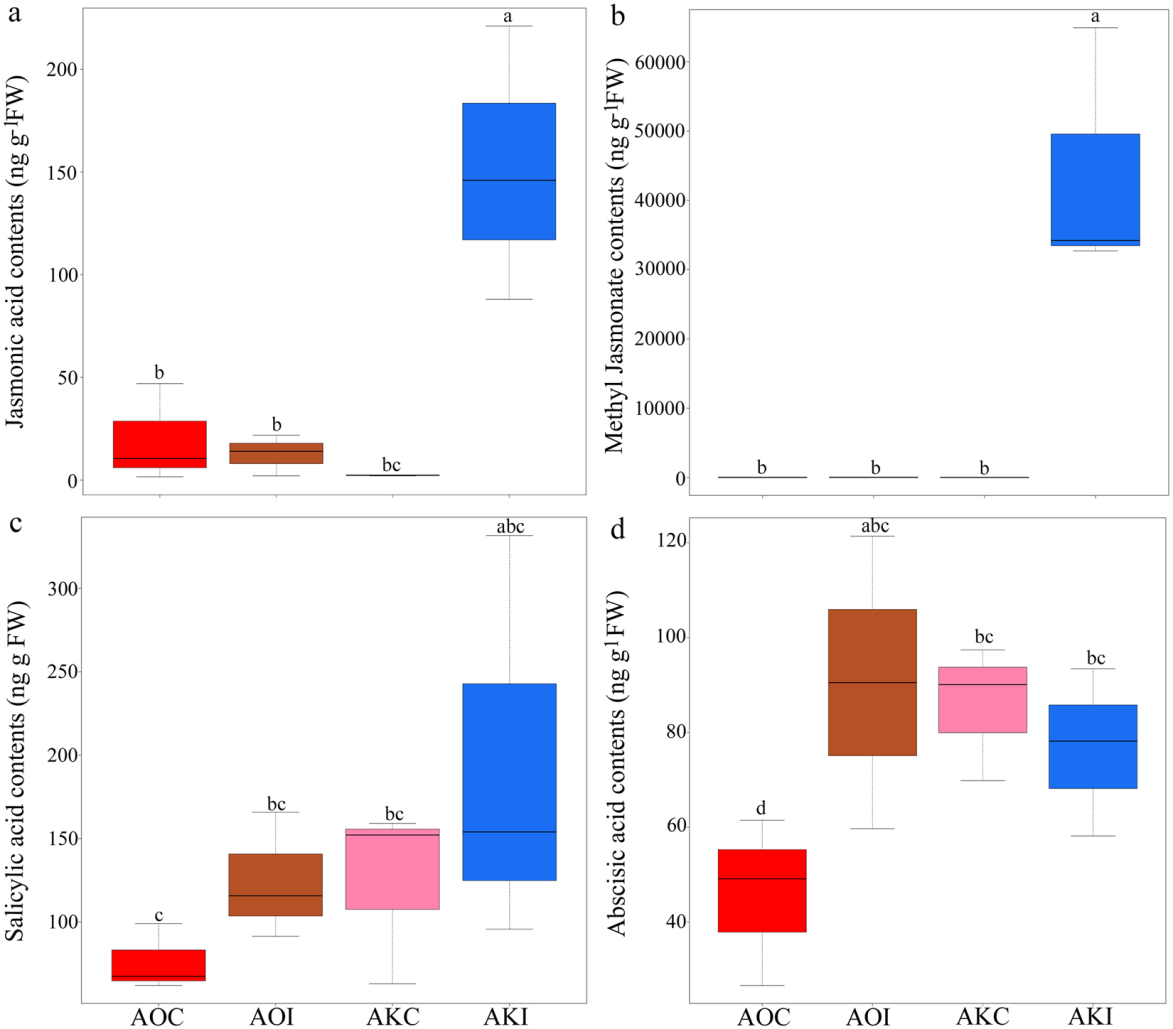
Box plot diagram showing the changes in (**a**) jasmonic acid (JA), (**b**) methyl jasmonate (MeJA), (**c**) salicylic acid (SA) and (**d**) abscisic acid (ABA) levels (ng g^−1^ FW) in *Asparagus kiusianus* inoculated with *Phomopsis asparagi* (AKI) and their relative levels in *A*. *officinalis* inoculated with *P*. *asparagi* (AOI). *A*. *kiusianus* and *A*. *officinalis* treated with sterile distilled water (AOC and AKC, respectively) served as controls. Values represent the maximum, third quartile, median, first quartile and minimum of three independent replicates (*n* = 3). Different letters indicate statistically significant difference at *P* < 0.05 according to Tukey’s honest significant difference (HSD) *post hoc* test.

Because JA biosynthesis and signaling-related genes were markedly elevated in AKI plants relative to AOC plants, we examined the JA, MeJA, SA and ABA levels in the AOC, AOI, AKC and AKI plants 24 h post-inoculation using LC-MS/MS (Fig. 7). JA and MeJA exhibited a significant (*P* < 0.001) increase at 65.92- and 14746.97-fold, respectively in AKI plants in comparison with AKC plants. However, there was no significant differences between AOI and AOC plants (Fig. 7a,b). SA levels exhibited non-significant increase in the two infected *Asparagus* species relative to control plants (Fig. 7c). In addition, ABA levels displayed a significant increase (*P* < 0.05) at 1.98-fold in AOI plants relative to AOC plants (Fig. 7d). However, non-significant differences were observed between AKI and AKC plants (Fig. 7d).

### Effects of *P*. *asparagi* on total protein content and stress-related enzyme activities in wild and cultivated *Asparagus* species

**Figure 8:**
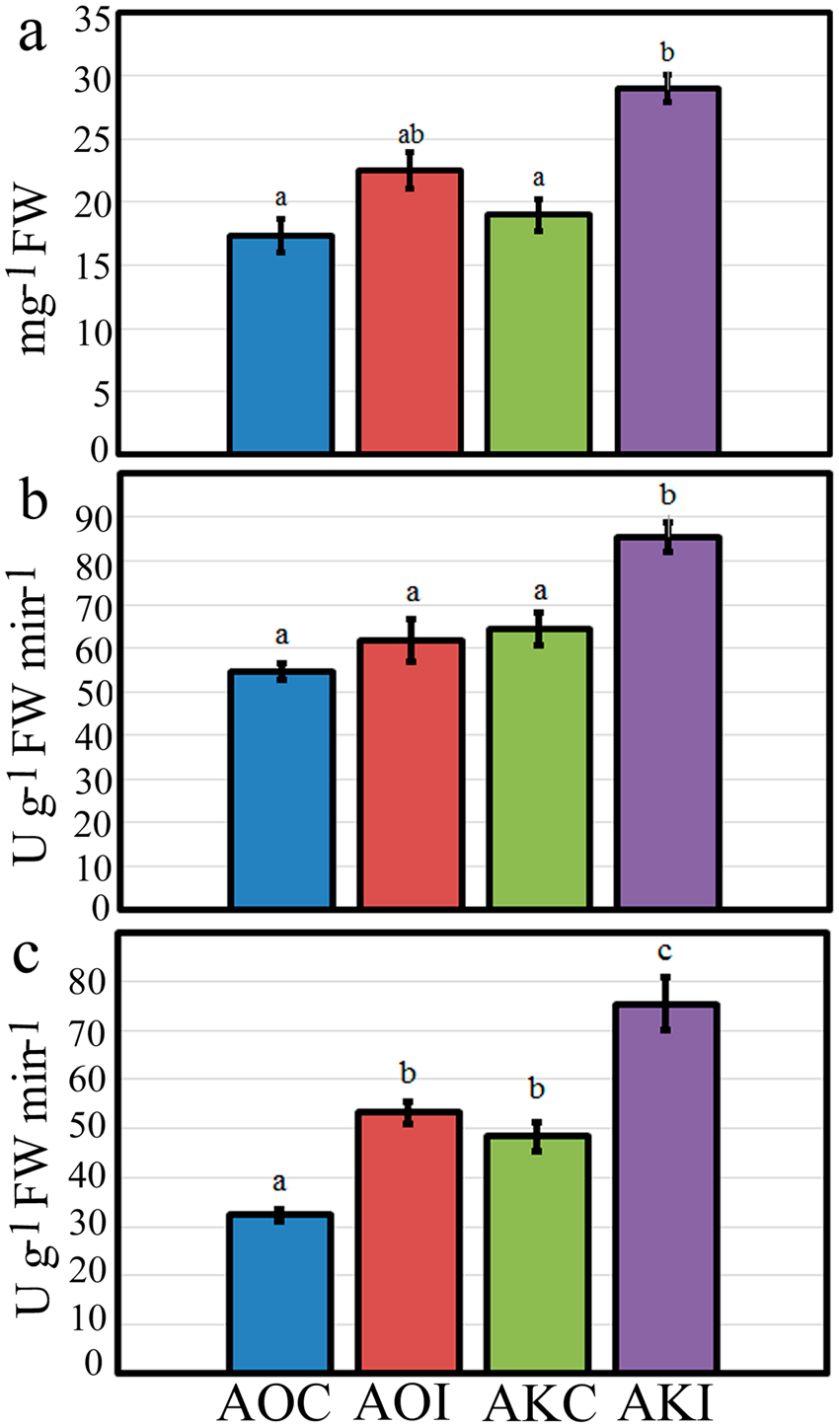
Accumulation of (**a**) total protein content, (**b**) peroxidase, and (**c**) catalase activities in *Asparagus officinalis* inoculated with *Phomopsis asparagi* (AOI) and *A*. *kiusianus* inoculated with *P*. *asparagi* (AKI). *A*. *officinalis* and *A*. *kiusianus* treated with sterile distilled water (AOC and AKC, respectively) served as controls. Values represent the mean ± standard error (SE) of three independent replicates (*n* = 3). Different letters indicate statistically significant difference at *P* < 0.05 according to Tukey’s honest significant difference (HSD) *post hoc* test.

In *A*. *kiusianus*, total protein content was significantly higher (1.52-fold increase; *P* < 0.008) in plants inoculated with *P*. *asparagi* (AKI) than in control plants (AKC). Conversely, in *A*. *officinalis*, although protein content was slightly higher in inoculated plants, no significant difference in total protein content was seen between inoculated (AOI) and control (AOC) plants (Fig. 8a). POX and CAT enzymatic assays were carried out to investigate the nature of the defence response in the two *Asparagus* species. No significant difference in POX activity was detected between inoculated (AOI) and control (AOC) *A*. *officinalis* plants. However, in *A*. *kiusianus*, POX activity was significantly higher (1.32-fold increase; *P* < 0.002) in inoculated (AKI) plants than in control (AKC) plants (Fig. 8b). Inoculation stimulated significant increases in CAT activity in both *A*. *officinalis* and *A*. *kiusianus*. CAT activity was 1.64-fold higher (*P* < 0.001) in AOI plants than in AOC plants and 1.55-fold higher (*P* < 0.001) in AKI plants than in AKO plants (Fig. 8c).

## Discussion

Asparagus stem blight, which is caused by *P*. *asparagi*, is a serious disease that affects asparagus production worldwide, and there is an urgent need to produce asparagus cultivars with strong resistance to this disease. After artificial inoculation with *P*. *asparagi*, *A*. *kiusianus*, a wild relative of cultivated *A*. *officinalis*, exhibited significantly (*P* < 0.001) reduced disease severity compared with *A*. *officinalis* (Fig. 1), consistent with a previous study examining disease in these *Asparagus* species^4^. These results suggest that wild *A*. *kiusianus* is a potential genetic reservoir for *Phomopsis* disease resistance improvement in cultivated *A*. *officinalis*. Indeed, interspecific hybridization between *A*. *kiusianus* and *A*. *officinalis* resulted in F_1_ hybrids with strong *Phomopsis* disease resistance^4^. However, the mechanisms underlying *Phomopsis* disease resistance in *A*. *kiusianus* remain unknown. Candidate *A*. *kiusianus* genes involved in *Phomopsis* disease resistance were identified by comparing transcription in susceptible *A*. *officinalis* and resistant *A*. *kiusianus* 24 h after infection with *P*. *asparagi*. Samples from inoculated and control *A*. *officinalis* and *A*. *kiusianus* plants were sequenced using the Illumina HiSeq 2500 platform, and *de novo* transcriptome assemblies were generated and compared. In total, 1,779 differentially expressed transcripts, 1,027 of which were up-regulated and 752 of which were down-regulated, were detected in the two *Asparagus* species in response to *P*. *asparagi* infection (Fig. 4). GO term enrichment and KEGG metabolic pathway analyses revealed that transcripts that were highly expressed in *A*. *kiusianus* and exhibited low expression in *A*. *officinalis* were enriched for metabolic pathways, biosynthesis of secondary metabolites, plant-pathogen interaction, protein kinase signaling and plant hormone biosynthesis and signaling transduction (Fig. 5 and Supplementary Table S3).

Expression of stress and defence response-related genes was strongly up-regulated in resistant wild *A*. *kiusianus* in comparison with susceptible *A*. *officinalis* (Figs 5 and 6) in response to *P*. *aspargi* infection. Up-regulation of *PR* genes, including *PR*-*1*, is a prerequisite for activation of systemic acquired resistance (SAR), which plays a central role in plant basal defence against pathogen infection^15, 16^. Plant chitinases and POXs, which are also classified as PR proteins, play direct roles in plant resistance by hydrolyzing fungal cell walls and oxidating phenolic residues in the infected tissues, respectively^17–19^. A similar pattern of up-regulation of stress and defence response-related genes was reported in leaves of *Withania somnifera* after stimulation of salicylic acid-induced defence mechanisms^19^. Up-regulation of *POX*, *chitinase*, and *PR* transcripts was also reported in *Fusarium*-resistant wheat (*Triticum aestivum*) compared with a susceptible genotype^20^. Similarly, rice (*Oryza sativa*) variety ‘Selenio’, which was resistant to *Fusarium fujikuroi*, exhibited up-regulation of several *PR* genes compared with the susceptible variety ‘Dorella’^21^. Our data and results from previous studies clearly suggest that POX, chitinase, and PR-1 play important roles in plant basal defenses and SAR induction during early plant responses to infection in resistant genotypes^22^. SAR is responsible for triggering host defence mechanisms to increase *de novo* expression of defense-related genes, leading to enhanced expression and *de novo* synthesis and accumulation of chitinases, POXs, and other pathogenesis-related proteins in uninfected tissues, thereby defending them against any further pathogen attack^23^. It is therefore likely that similar defence pathways are involved in responses to *P*. *asparagi* infection in wild *A*. *kiusianus*.

Our results also indicate a possible role for genes encoding ribosomal proteins (RPLs) in *Asparagus* defence mechanisms. Several genes encoding RPLs were strongly up-regulated in inoculated *A*. *kiusianus* (AKI) plants and suppressed in inoculated *A*. *officinalis* (AOI) plants (Fig. 5d). RPLs have established roles in facilitating protein synthesis and preserving the stability of the ribosomal complex^24^. The involvement of RPLs in plant defence was recently reported in non-host resistance against bacterial pathogenic attack in *Nicotiana benthamiana* ^25^. The study suggested that RPL12 and RPL19 played substantial roles in the development of non-host disease resistance through the induction of the hypersensitive response (HR)^25^. Similarly, strong up-regulation of 80% of rice RPL-related genes was observed in response to exposure to *Xanthomonas oryzae* ^24^. The putative promoter regions of these RPL-related genes carry *cis*-elements that respond specifically to stress, suggesting that genes in the RPL family might be useful targets in strategies to develop stress tolerance in rice and other crops^24^. Large-scale analysis of differential gene expression in coffee (*Coffea arabica*) genotypes that were resistant or susceptible to leaf miner revealed up-regulation of RPL-encoding genes in the resistant genotype compared with the susceptible genotype^26^. Our results indicated that genes encoding RPLs may be involved in disease resistance in *A*. *kiusianus* in response to *P*. *asparagi* exposure, and that this response may be species-specific. Further molecular and biochemical analyses of RPLs identified in this study will be needed to understand the functional role of RPLs in the *Asparagus* defence mechanism.

In the present study the expression level of several JA biosynthesis-related genes including *PLDα1*, *LOX1*, *OPR and JIP*-*23 KD* was elevated in the resistant wild *A*. *kiusianus* in comparison with susceptible *A*. *officinalis* in response to *P*. *aspargi* infection (Figs 5 and 6). *PLD* and *LOX* genes are necessary for the initial steps of JA biosynthesis in *Arabidopsis* ^27^. PLD release linolenic and α-linolenic acids from chloroplast membranes and these substrates are subsequently catalyzed by LOX, leading to the formation of hydroperoxy octadecadienoic acids^27^. During the early stage of plant-pathogen interaction the activation of PLD and LOX is essential for the production of important defense signaling molecules, such as oxylipins and JA which has been shown to modulate the activity of variety of proteins involved in defense signaling^28^. RNA-Seq analysis of *F*. *fujikuroi*- resistant rice variety ‘Selenio’ showed up-regulation in *PLD* and *LOX* genes in comparison with susceptible rice ‘Dorella’, indicating a crucial role of JA pathway in the resistance of rice to *F*. *fujikuroi* infection^21^. JA signaling has broadly been associated to the defense against necrotrophic pathogens, inducing the accumulation of secondary metabolites and PR proteins^29^. However, recent studies have revealed that certain biotrophic fungal species can also trigger JA-mediated responses^29^. In this context, *Phomopsis* pathogens are nectrotrophic at least for the latent phase of infection and are therefore named hemi-biotrophs^30^. In *A*. *kisuainus* a substantial increase in *OPR* expression was observed (Figs 5 and 6). OPR catalyzes the final step in JA biosynthesis by reducing 12-oxophytodienoic acid to 3-oxo-2(2′-pentenyl)-cyclopentane-1-octanoic acid^28^. *OPR* was previously proposed as a disease resistance marker in tomato (*Solanum lycopersicum*)^31^. A cleaved amplified polymorphic sequences (CAPS) marker derived from tomato *OPR* showed putative co-segregation with a potato (*S*. *tuberosum*) quantitative trait locus (QTL) for late blight disease resistance. Likewise, gene expression profile of JA biosynthesis-related genes (*LOX*, *AOC* and *OPR*) in *Plasmopara viticola* disease resistance and susceptible grapevine (*Vitis vinifera*) cultivars, revealed a strong up-regulation of these genes in the resistant cultivar in comparison with susceptible cultivar^32^. Additionally, expression of *OPR* and *LOX* was substantially higher in a *Fusarium* head blight-resistant wheat variety ‘Wangshuibai’ than in a susceptible variety ‘NAUH117’ 24–48 h after infection^33^. These results suggest that *OPR* and *LOX* have potentially important roles in the early defence response in wheat against *Fusarium* head blight^33^. Another JA biosynthesis-related gene, *JIP*-*23 KD*, was expressed at substantially higher levels in inoculated *A*. *kiusianus* (AKI) plants than in AKC plants. In *A*. *officinalis*, *JIP*-*23 KD* exhibited reduced expression in inoculated plants (AOI) compared with control plants (AOC) (Fig. 5d). An early study examining the effect of JA on the interactions between barley (*Hordeum vulgare*) and powdery mildew suggested a possible role for JIP-23 KD in cell wall modification or in pathogen defence^34^. However, the specific roles of JIP-23 KD in pathogen defence remain to be determined^34^. Recently, *JIP*-*23 KD* was shown to be up-regulated in cadmium-tolerant barley genotype compared with a cadmium-susceptible genotype^35^. Collectively, these results suggest that JIP-23 KD may have a role in plant stress responses. The up-regulation of *PLDα1*, *LOX1*, *OPR* and *JIP*-*23 KD* genes in *A*. *kiusianus* inoculated with *P*. *asparagi* suggests that JA signal transduction may play a crucial role in *Phomopsis* disease resistance in *A*. *kiusianus*. Phytohormone analysis revealed a significant increase in JA and MeJA contents in wild-resistant *A*. *kiusianus* relative to cultivated-susceptible *A*. *officinalis* in response to *P*. *asparagi* infection (Fig. 7), providing an additional support for our hypothesis regarding the important role of JA-dependent signaling pathway in the *P*. *asparagus* disease resistance.

The fluctuation in POX and CAT activities between resistance and susceptible genotypes has been previously reported^36^. Therefore, stress-response enzyme activities were assessed in the present study. A significant (*P* < 0.002) increase in POX activity was seen in *A*. *kiusianus* plants after *P*. *asparagi* infection (AKI) compared with control plants (AKC). No significant difference was seen in POX activity between inoculated (AOI) and control (AOC) *A*. *officinalis* plants (Fig. 7). This suggested that POX played a substantial role in *A*. *kiusianus* defence via suppression of *P*. *asparagi* spread. The accumulation pattern of POX in inoculated *A*. *kiusianus* plants correlated with the up-regulation of several POX-related genes in the RNA-Seq data (Fig. 5c). Similar findings were reported in cabbage (*Brassica oleracea* var. *capitata*), where strong POX activities and up-regulation of *POX*-related genes was seen in cabbage resistant to black rot disease^37^. CAT activity was significantly (*P* < 0.001) induced by *P*. *asparagi* inoculation in both *A*. *kiusianus* (AKI) and *A*. *officinalis* (AOI) (Fig. 7c). Similar findings were previously reported in *A*. *officinalis* inoculated with *P*. *asparagi* ^2^: CAT activities increased at all time points after infection whereas POX activities increased only initially, and declined thereafter^2^. These data indicate that CAT is involved in basal defence mechanisms in both resistant and susceptible *Asparagus* species.

This is the first study to examine the molecular responses of *Asparagus* species to *P*. *asparagi* infection. RNA sequencing was used to identify DEGs in resistant wild *A*. *kiusianus* and susceptible cultivated *A*. *officinalis* 24 h after infection with *P*. *asparagi*. Functional annotation and KEGG pathway analysis showed that the group of up-regulated genes in *A*. *kiusianus* was enriched for metabolic pathways, biosynthesis of secondary metabolites, plant-pathogen interaction, transcriptional regulation, protein kinase signaling, phenylpropanoid and hormone biosynthesis and signaling. Results from RNA-Seq data and qRT-PCR were correlated, confirming the reliability of the transcriptome data. Activity of the stress-related enzyme POX was elevated in *A*. *kiusianus* compared with *A*. *officinalis*. Overall, comparative transcription profiling provided valuable insights into the mechanisms underlying *Phomopsis* disease resistance in *Asparagus*. These findings will be valuable in the future development of disease-resistant asparagus varieties. The RNA-Seq datasets generated in this study will be mined for sequence variations associated with gene structure and function, which will facilitate genetic trait mapping and marker-assisted selection in asparagus breeding programs.

## Methods

### Plant materials

Cultivated *A*. *officinalis* ‘Mary Washington 500W’ (AO0060 strain) and wild *A*. *kiusianus* (AK0501 strain) for RNA-Seq analysis were grown under standard greenhouse conditions (25 ± 2 °C and 14 h light/10 h dark) at Kagawa Prefectural Agricultural Experiment Station (Kagawa, Japan). Male *A*. *kiusianus* and female *A*. *officinalis* were kept to grow for 4-year-old in a commercial soil consisted of loam, well-drained soil, leaf mold and garden soil with fertilizers (2:1:1:2 and 2:1:1:4 for *A*. *kiusianus* and *A*. *officinalis*, respectively v:v) containing 50 mg N kg^−1^, 500 mg P kg^−1^, and 100 mg K kg^−1^ [pH 6–6.5, water-holding capacity ~70%, Nippi Engei Baido No.1 (Nihon Hiryo Co. Ltd)] as a root-supporting medium (Supplementary Fig. S2).

### Inoculation of *A*. *officinalis* and *A*. *kiusianus* with *P*. *asparagi* for RNA-Seq analysis

Plants were artificially inoculated with spores of *P*. *asparagi* at a final concentration of 10^7^ CFU ml^−1^ according to the vinyl cotton (VC) method^38^ (Supplementary Fig. 2). Minimal methodological modifications were made: silicone tubes were used instead of vinyl tubes and water-retentive polyester fiber sheeting was used instead of cotton. Stems from three independent biological replicates (n = 3) were harvested 24 h after infection, immediately frozen in liquid nitrogen, and stored at −80 °C until used. Plants treated with SDW under the same conditions served as a control.

### Determination of stem blight disease severity in cultivated and wild *Asparagus* species

In a separate experiment, *A*. *officinalis* and *A*. *kiusianus* plants cultivated and inoculated as described above were kept for 3 weeks after inoculation and disease severity on the infected stems was recorded. Disease severity was scored on a 1–5 scale, where 1 = healthy and 5 = heavily infected. Disease severity percentage was calculated based on the following formula:

1 $$\begin{array}{l}{\rm{Disease}}\,{\rm{severity}}\,{\rm{percentage}}\\ \begin{array}{rcl} & = & [({\rm{Sum}}\,{\rm{of}}\,{\rm{all}}\,{\rm{disease}}\,{\rm{ratings}})/({\rm{Total}}\,{\rm{number}}\,{\rm{of}}\,{\rm{ratings}}\\  &  & \times {\rm{Maximum}}\,{\rm{disease}}\,{\rm{grade}})]\times 100\end{array}\end{array}$$

### RNA extraction, cDNA library construction, and Illumina sequencing

Total RNA was extracted from three independent biological replicates (n = 3) using a QIAGEN RNeasy Plant Mini Kit (QIAGEN Sciences, Maryland, USA) following the manufacturer’s instructions. The quality and quantity of RNA were assessed using 2% agarose gel electrophoresis and an Agilent 2100 Bioanalyzer (Agilent Technologies Inc., USA) with a minimum RNA integrated number of eight. Sequencing libraries were generated using a TruSeq Stranded RNA Sample Preparation Kit (Illumina). The cDNA libraries were sequenced using an Illumina HiSeq 2500 instrument (Illumina Inc., USA), and both ends of the inserts were sequenced.

### RNA-Seq data processing and *de novo* transcriptome assembly

The quality of the raw sequences was inspected using the open-source software FastQC (www.bioinformatics.babraham.ac.uk/projects/fastqc). After quality checking, adapter sequences were removed by Cutadapt v.1.3^39^, and low-quality (phred score < 30) and short (length < 50 bp) reads were trimmed by Sickle v.1.200 (www.github.com/najoshi/sickle). The resulting high-quality trimmed and size-selected reads were then *de novo* assembled using Trinity package v.2.0.6^40, 41^. The resulting assembled unique transcripts were annotated by alignment against the NCBI nucleotide (nt) database (http://www.ncbi.nlm.nih.gov), using Blastn algorithms with a significance threshold of E-value Ie-3. The likely open reading frame (ORF) for each transcript was extracted using the TransDecoder program (www.transdecoder.sourceforge.net). Predicted ORFs were searched against the UniProt (Swiss-Prot) database using Blastp with a significance threshold of E-value Ie-6^42^. To annotate transcripts with GO terms, the top hit from the NCBI nt database for each transcript was submitted to the Blast2GO platform^43^, and GO terms for each transcript were retrieved based on the relationship between gene names and GO terms.

### Differential gene expression analysis

The expression level of each transcript was measured with a FPKM (fragments per kilobase of exon per million fragments mapped) method. FPKM values were calculated using RSEM v.1.2.19^44^. Genes that were differentially expressed (DEGs) between *A*. *officinalis* and *A*. *kiusianus* 24 h after inoculation with *P*. *asparagi* were determined using BESeq R Bioconductor^45, 46^. Genes were classified as DEG if they exhibited ≥2-fold changes between the two samples with statistical significance adjusted-*P* < 0.05^47^.

### KEGG pathway analysis

The metabolic pathways of the up-regulated DEG in *A*. *kiusianus* in response to *P*. *aspargi* infection was constructed based on KEGG database. Initially, all the DEG uniprot ID was converted into KEGG ID by using retrieve/ID mapping (http://www.uniprot.org/uploadlists). Further, the KO number for each metabolic pathway was obtained by using KEGG mapper web-based tool (http://www.genome.Jp/keg/tool/map_pathway2.html).

### Experimental validation of candidate gene expression by qRT-PCR

To confirm DEG results, qRT-PCR was performed on six selected up-regulated disease resistance candidate genes from *A*. *kiusianus* libraries. Details of gene annotations and primer sets are shown in Supplementary Table S2. Gene-specific primers and internal standard primers were designed using the aligned sequence of *A*. *officinalis* and *A*. *kiusianus* cDNA obtained from the Illumina RNA-Seq data. First, real time reverse transcription-PCR (RT-PCR) was carried out using a Takara PCR Thermal Cycler and their PCR products were checked using 2% agarose gel electrophoresis. Second, qRT-PCR was performed using a SYBR Green Supermix Kit (Bio-Rad Laboratories, Inc.) with a Mini Option Real-Time PCR system (Bio-Rad). Relative expression values were calculated using the 2^−ΔΔCt^ method, with elongation factor as an internal standard. RNA pools used in the RT-PCR and qRT-PCR analyses were extracted from samples used for RNA-Seq. All reactions were performed with three biological replicates.

### Quantification of plant hormones

For analysis of JA, MeJA, SA and ABA, 100 mg from AKC, AKI, AOC and AOI stem was homogenized separately in liquid nitrogen and placed in 5 ml of 80% methanol containing 0.1% acetic acid. [^2^H_2_]JA, [^2^H_2_]MeJA (Tokyo Chemical Industry), [^2^H_4_]SA (Cambridge Isotope Laboratories) and [^2^H_6_]ABA (OlchemIm) were added to the extracts to serve as internal standards. After overnight extraction at 4 °C, solids were separated by centrifugation and re-extracted for 30 min in 5 ml of the same extraction solution. The extracts were combined, and passed through BOND ELUT C18 column (500 mg, Agilent Technologies), equilibrated with 1% acetic acid. The eluate was evaporated and dissolved in water:methanol:acetic acid (89.9:10:0.1, v-v:v) and analyzed by LC-MS/MS system. The LC-MS/MS system consisted of a Prominence 20A Series HPLC (Shimadzu) equipped with a 3200 QTrap LC/MS/MS System (AB Sciex), using an electrospray interface. For quantification of JA, SA and ABA, the purified samples were injected onto a Cadenza CD-C18 column (3 µm, 150 × 3.0 mm; Imtakt) at 45 °C and eluted at a flow rate of 0.2 ml min^−1^. Hormones were separated with a gradient of mobile phase A (water:methanol:acetic acid, 89.9:10:0.1) and B (methanol). The initial conditions were 100% A, maintaining for 2 min, changing linearly to 40% A and 60% B in 5 min, changing to 100% B in 10 min, and finally maintained at 100% B for 6 min. The column was equilibrated with the starting composition of the mobile phase for 12 min. For quantification of MeJA, the purified samples were injected onto a Shim-pack XR-ODS column (2.2 μm, 75 × 2.0 mm; Shimadzu). The mobile phases and gradient conditions were described above. Quantification was obtained by multiple reaction monitoring (MRM) of the selected precursor ions and a specific product ions as described in Supplemental Table S4.

### Preparation of crude enzyme extract

Stem tissue (200 mg) was ground to a fine powder in liquid nitrogen using a pre-cooled mortar and pestle, and 2 ml of extraction buffer [0.2 M phosphate buffer (pH 7.2), 0.1 mM EDTA, 1 mM DTT, and 2 U protease inhibitor cocktail] was added. The macerated suspension was centrifuged at 10,000 rpm for 5 min at 4 °C. The supernatant was collected and used as the source of enzyme. Total protein content of the crude enzyme extract was determined using a Bradford assay^48^.

### Determination of stress-related enzyme activities

POX activity was estimated using a UV/Vis Beckman DU 730 spectrophotometer (Beckman Coulter Inc. California, USA) as described previously^49^. The reaction mixture, which consisted of 0.8 ml of 0.2 M phosphate buffer (pH 7.2), 1 ml of 15 mM guaiacol, 1 ml of 3 mM hydrogen peroxide, and 0.2 ml of crude enzyme extract, was incubated at room temperature for 3 min. Absorbance of the colored product was monitored at 470 nm. POX activity expressed as ∆470 g^−1^ fresh weight (FW) min^−1^ was calculated using the following formula:

2 $$\begin{array}{rcl}{\rm{U}}/{\rm{ml}} & = & [{\rm{Change}}\,{\rm{in}}\,{\rm{absorbance}}\,{{\rm{\min }}}^{-1}\times {\rm{Reaction}}\,{\rm{mixture}}\,{\rm{volume}}\,({\rm{ml}})\\  &  & \times {\rm{Dilution}}\,{\rm{factor}}]/[{\rm{\varepsilon }}470\times {\rm{Enzyme}}\,{\rm{extract}}\,{\rm{volume}}\,({\rm{ml}})]\end{array}$$

CAT activity was determined spectrophotometrically at 240 nm as described previously^50^. CAT activity expressed as ∆240 g^−1^ FW min^−1^ was calculated using the following formula, modified with hydrogen peroxide coefficient *ε*240:

3 $$\begin{array}{rcl}{\rm{U}}/{\rm{ml}} & = & [{\rm{Change}}\,{\rm{in}}\,{\rm{absorbance}}\,{\min }^{{\rm{-}}1}\times {\rm{Reaction}}\,{\rm{mixture}}\,{\rm{volume}}\,({\rm{ml}})\\  &  & \times {\rm{Dilution}}\,{\rm{factor}}]/[{\rm{\varepsilon }}240\times {\rm{Enzyme}}\,{\rm{extract}}\,{\rm{volume}}\,({\rm{ml}})]\end{array}$$

## Electronic supplementary material

Supplementary information
