# Supplementary material for: Comparative de novo transcriptome profiles in Asparagus officinalis and A. kiusianus during the early stage of Phomopsis asparagi infection
Source: Sci Rep. 2017 Jun 1;7:2608. doi: 10.1038/s41598-017-02566-7 (PMC5453997; doi:10.1038/s41598-017-02566-7)

Comparative *de novo* transcriptome profiles in *Asparagus officinalis* and *A. kiusianus* during the early stage of *Phomopsis asparagi* infection.

Mostafa Abdelrahman[1,2,*], Naoyuki Suzumura[1], Mai Mitoma[1], Satoshi Matsuo[3], Takao Ikeuchi[4], Mitsutaka Mori[4], Kyoko Murakami[4], Yukio Ozaki[5], Masaru Matsumoto[6], Atsuko Uragami[7], & Akira Kanno[1,*]

[1]Graduate School of Life Sciences, Tohoku University, Katahira 2-1-1, Aoba-ku, Sendai 980-8577, Japan.

[2]Botany Department, Faculty of Science, Aswan University, Aswan 81528, Egypt.

[3]Institute of Vegetable and Floriculture Science, National Agriculture and Food Research Organization (NARO), 360 Kusawa, Ano, Tsu, Mie 514-2392, Japan.

[4]Kagawa Prefectural Agricultural Experiment Station, 1534-1 Ayagawa, Ayauta, Kagawa 761-2306, Japan.

[5]Faculty of Agriculture, Kyushu University, Fukuoka 811-2307, Japan.

[6]Institute of Tropical Agriculture, Kyushu University, Fukuoka 812-8581, Japan.

[7]National Agriculture and Food Research Organization, Tsukuba, 305-8519, Japan.

Correspondence and requests for materials should be addressed to Mostafa Abdelrahman (email: meettoo2000@ige.tohoku.ac.jp) or Akira Kanno (email: kanno@ige.tohoku.ac.jp)

Supplementary Table S1: Summary of data quality of *Asparagus* transcriptome sequences. The data describe the total raw read sequences, trimmed reads, and assembled transcripts with total numbers of detected open reading frames (ORFs).

Accession	Library	Raw reads	Clean reads	Q30 (%)	Contigs	ORF
AO0060	*A. officinalis* control	106,020,403	94,440,525	96.6	206,164	60.299
AO0060	*A. officinalis* infected	113,444,927	101,235,105	96.7	206,164	60.299
AK0501	*A. kiusianus* control	100,269,889	90,926,718	96.9	213,950	63.284
AK0501	*A. kiusianus* infected	104,006,596	94,362,439	97.1	213,950	63.284

Supplementary Table S2: List of gene annotations and primer pairs used for real-time reverse transcription PCR (RT-PCR) and quantitative real-time reverse transcription PCR (qRT-PCR) experiments. The *elongation factor 1-alpha* (*eEF1A*) gene was used as the reference gene for internal control for normalization.

Gene annotation	Primer pairs
*12-oxophytodienoate reductase*11	F: 5´ CCATGTAGAGACCGAGAGC-3´<br>R: 5´ GCTGACAGAGTTGGGATAAG-3´
chitinase-6	F: 5´ GCACTCCTGGCTGTTGATC-3´<br>R: 5´ CTAGTCTCGTTCAAGGCGG-3´
40S ribosomal protein S11	F: 5´ TGATTTCTTCGAGCAAAGGAACACC-3´<br>R: 5´ CCGCTCTCCAATCTCTCGCA-3´
blue copper protein	F: 5´ TCGATCTCGACCTTCATCC-3´<br>R: 5´ CGACAAGCAATCCGATAGC-3´
CYP71A1	F: 5´ CTCGTAGGCAATCTCTGCTG-3´<br>R: 5´ GCAACCTCCACCAACTAGG-3´
aspartic proteinase nepenthesin	F: 5´ GGGAGAGATTGACAAAGGG-3´<br>R: 5´ GGCAGCAACCTAATCTGG-3´
pathogenesis-related protein 1-like	F: 5´ GCAGGTGACAAATACTCCG<br>R: 5´ AGGGTCATGAATGTAGGCAC
peroxidase4	F: 5´ TCTCTCCGGTGAAGCTAGAG<br>R: 5´ GACGATCAAATCAGTGGTCC
WRKY41	F: 5´ GTGCTAGTGAACGAATTGGG<br>R: 5´ CAAGACAAGCGAGTATGTCG
phospholipase D alpha 1	F: 5´ GTGACTTCCGAGCAGTTCTG<br>R: 5´ CCATTCGAAAGATGCGAG
jasmonate induced protein-23 KD	F: 5´ CTGAAAGCAATGAGCCAGTAC<br>R: 5´ CCTTGACGTATTTGAGTGGAG
s-methyltransferase 1	F: 5´ CGTTGTCTGATCTCCAATCC<br>R: 5´ GCTTTCGTTGACACTCAGC
metallothionin 2	F: 5´ CAACATGTACGCTGATTTCG<br>R: 5´ CTTGCATCCATTTTCAGCTC
cationic peroxidase SPC4-like	F: 5´ TCCATCAAACTCGACACCAC<br>R: 5´ GAAGGACTTGTTGAGGGTGG
phloem protein 2-like A1	F: 5´ CATGCCCAAGAACGAGTG<br>R: 5´ CACACAACAGCAGAACATCAC
elongation factor 1-alpha	F: 5´ CTGGCCAGGGTGGTTCATGAT-3´<br>R: 5´ TAAGTCTGTTGAGATGCACC-3´

Supplementary Table S3: Enriched KEGG pathways of differentially up-regulated genes in *A. kiusianus* in response to *Phomopsis asapargi* infection.

Pathway	% of up-regulated genes	Pathway ID
Metabolic pathways	18.18	map01100
Biosynthesis of secondary metabolites	11.11	map01060
Plant-pathogen interaction	6.06	map04626
MAPK signaling pathway	5.05	map04010
Plant hormone signal transduction	5.05	map04057
Carbon metabolism	4.04	map01200
Biosynthesis of amino acids	4.04	map01230
Starch and sucrose metabolism	4.04	map00500
Purine metabolism	3.03	map00230
Glutathione metabolism	3.03	map00480
Fructose and mannose metabolism	3.03	map00051
Amino sugar and nucleotide sugar metabolism	3.03	map00520
Alpha-Linolenic acid metabolism	2.02	map00592
Zeatin biosynthesis	2.02	map00908
Selenocompound metabolism	2.02	map00450
Ribosome	2.02	map03010
Phenylpropanoid biosynthesis	2.02	map00940
Peroxisome	2.02	map04146
Pentose phosphate pathway	2.02	map00030
Glycolysis / Gluconeogenesis	2.02	map00010
Glycerophospholipid metabolism	2.02	map00564
Inositol phosphate metabolism	1.01	map00562
Flavonoid biosynthesis	1.01	map00941
Terpenoid backbone biosynthesis	1.01	map00900
Sulfur metabolism	1.01	map00920
Steroid biosynthesis	1.01	map00140
Protein processing in endoplasmic reticulum	1.01	map04141
Nitrogen metabolism	1.01	map00910
Glycerolipid metabolism	1.01	map00561
Arginine and proline metabolism	1.01	map00330
Vitamin B6 metabolism	1.01	map00750
Cysteine and methionine metabolism	1.01	map00270

Supplemental Table S4: Parameters for measurements of jasmonic acid (JA), methyl jasmonate (MeJA), salicylic acid (SA) and abscisic acid (ABA) by liquid chromatography-tandem mass spectrometry (LC-MS/MS) analysis. DP, declustering potential; EP, entrance potential; CEP, collision cell entrance potential; CE, collision energy; CXP, collision cell exit potential.

Compound	Polarity	MS/MS transition (*m/z*)	DP (V)	EP (V)	CEP (V)	CE (V)	CXP (V)
JA	-	209.0/59.0	-20	-8.5	-16	-20	0
[$^2H_2$]JA	-	211.0/58.9	-25	-4	-16	-22	0
MeJA	+	225.2/133.2	26	11	16	19	4
[$^2H_2$]MeJA	+	227.2/153.2	26	9	16	17	4
SA	-	137.1/93.1	-30	-4	-8	-24	0
[$^2H_4$]SA	-	140.9/96.9	-20	-9	-10	-44	0
ABA	-	263.1/153.0	-25	-4.5	-20	-14	-2
[$^2H_6$]ABA	-	269.1/159.1	-30	-3.5	-20	-20	-2

Supplementary Figure S1: Gel electrophoresis of reverse transcriptase real-time PCR (RT-PCR) in *Asparagus* species under control and inoculated conditions. RT-PCR of selected candidate gene expression levels in *A. kiusianus* and *A. officinalis* treated with sterile distilled water (AKC and AOC, respectively) and inoculated with *Phomopsis asparagi* (AKI and AOI, respectively). Elongation factor 1-alpha served as an internal control.

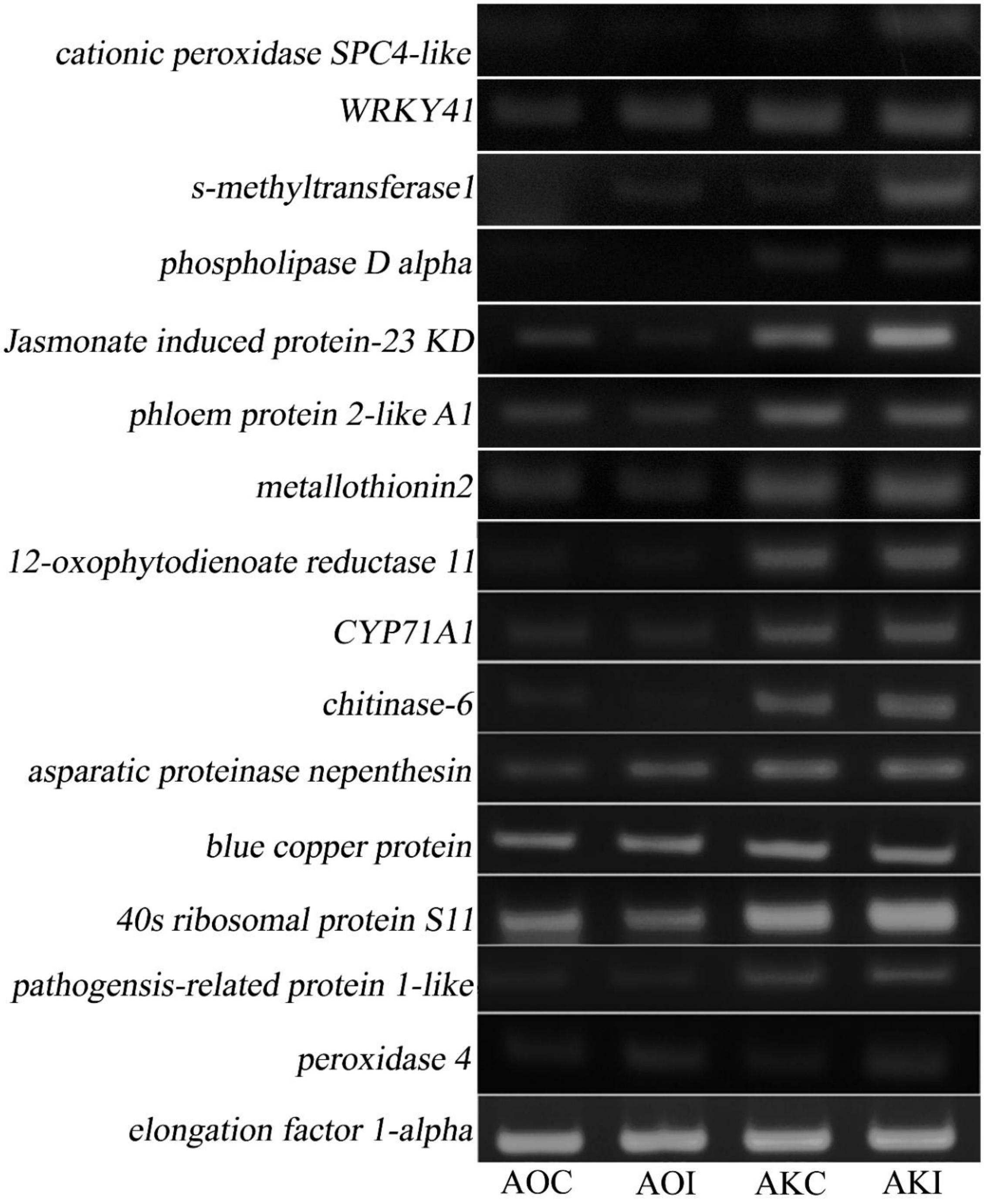

Supplementary Fig. S2: ***Asparagus officinalis*** **and** ***A. kiusianus*** **plants growing in the greenhouse under controlled environment conditions.** Plants were inoculated with spores of *Phomopsis asparagi* using vinyl cotton method

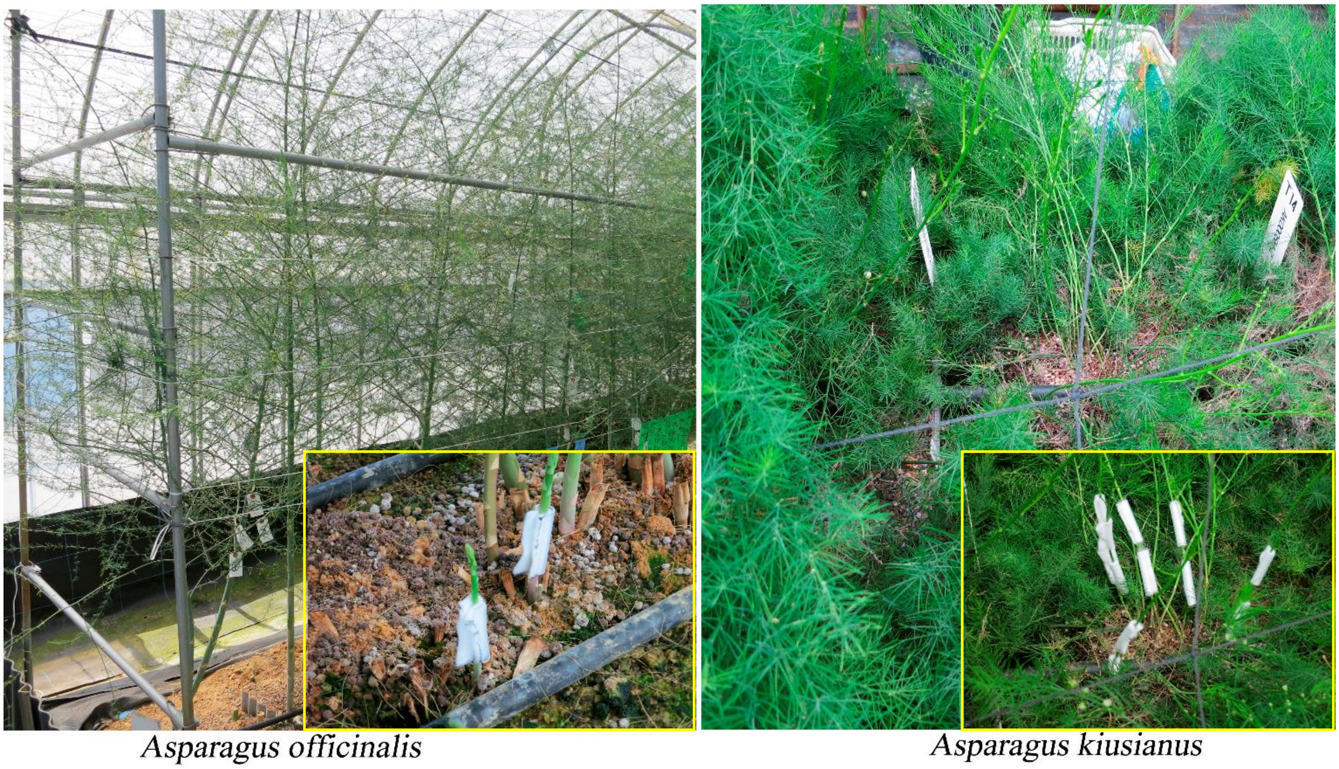

Supplement: Supplementary file 1 — Supplementary information [file 41598_2017_2566_MOESM1_ESM.pdf]
